# Novel GluN2B selective NMDA receptor antagonists: relative configuration of 7-meth­oxy-2-methyl-2,3,4,5-tetra­hydro-1*H*-3-benzazepin-1-ols

**DOI:** 10.1107/S2056989016005843

**Published:** 2016-04-15

**Authors:** Bastian Tewes, Bastian Frehland, Roland Fröhlich, Bernhard Wünsch

**Affiliations:** a Institut für Pharmazeutische und Medizinische Chemie der Universität Münster, Corrensstrasse 48, D-48149 Münster, Germany; b Organisch-chemisches Institut der Westfälischen Wilhelms-Universität Münster, Corrensstr. 40, D-48149-Münster, Germany; c Cells-in-Motion Cluster of Excellence (EXC 1003 – CiM), Universität Münster, Germany

**Keywords:** crystal structure, NMDA receptor antagonists, GluN2B antagonists, ifenprodil analogs, tetra­hydro-3-benzazepines, relative configuration, conformational restriction

## Abstract

The title compounds, C_22_H_29_NO_2_ (**3**) and C_22_H_29_NO_2_ (**4**) [systematic names: (1S*,2R*)-7-meth­oxy-2-methyl-3-(4-phenyl­but­yl)-2,3,4,5-tetra­hydro-1*H*-3-benzazepin-1-ol and (1R*,2R*)-7-meth­oxy-2-methyl-3-(4-phenyl­but­yl)-2,3,4,5-tetra­hydro-1*H*-3-benzazepin-1-ol, are diastereomers with the relative configuration of the adjacent hydroxyl and methyl groups at the seven-membered azepine ring being *trans* in (**3**) and *cis* in (**4**). In the crystals the orientation of these groups is *−anti-periplanar* (**3**) and *+syn-clinal* (**4**). In both cases, the crystals studied proved to be of a racemic mixture, with relative configurations (R*,S*)-**3** and (R*,R*)-**4**. In both compounds, the seven-membered azepine ring has a chair-like conformation, and the 4-phenyl­butyl side chain adopts a extended conformation in (R*,S*)-**3**, but a twisted conformation in (R*,R*)-**4**. In the crystal of (S*,R*)-**3**, mol­ecules are linked *via* C—H⋯O hydrogen bonds, forming slabs parallel to the *ac* plane. In the crystal of (R*,R*)-**4**, mol­ecules are linked *via* O—H⋯N hydrogen bonds, forming chains propagating along the *c-*axis direction. The chains are linked by C—H⋯O hydrogen bonds, forming slabs parallel to the *ac* plane.

## Abstract 2

Introduction of the flexible amino­alcohol substructure of ifenprodil into a more rigid ring system resulted in 2-methyl-2,3,4,5-tetra­hydro-1*H*-3-benzazepin-1-ols, (**3**) and (**4**), showing GluN2B affinity in the low nanomolar range. The chiral pool synthesis starting with (*R*)-alanine led to two diastereomers. The relative configuration of the benzazepines (**3**) and (**4**), that crystallized as racemates, was determined to be (S*,R*)-**3** and (R*,R*)-**4**.

## Chemical context   

(*S*)-Glutamate is the most important excitatory neurotransmitter in the central nervous system. It inter­acts with different metabotropic and ionotropic glutamate receptors. The NMDA (*N*-methyl-d-aspartate) receptor is one of three ionotropic receptors, which control the influx of cations, in particular Na^+^ and Ca^2+^ ions, into neurons (Bräuner-Osborne *et al.*, 2000 ▸; Kew & Kemp, 2005 ▸). Physiological activation of the NMDA receptor is associated with processes like learning and memory. However, over-activation of the NMDA receptor is connected with damage of neuronal cells leading finally to neuronal cell death. Therefore, inhibition of the NMDA associated ion channel could be useful for the treatment of traumatic brain injury, cerebral ischemia, neuropathic pain, depression and neurodegenerative disorders like Alzheimer’s and Parkinson’s disease (Bräuner-Osborne *et al.*, 2000 ▸; Kew & Kemp, 2005 ▸; Paoletti *et al.*, 2013 ▸; Wu & Zhou, 2009 ▸).

The amino­alcohol ifenprodil inhibits selectively NMDA receptors containing GluN2B subunits (Williams, 2001 ▸; Borza & Domány, 2006 ▸; Layton *et al.*, 2006 ▸; Karakas *et al.*, 2011 ▸). In order to improve the affinity, selectivity and metabolic stabil­ity of ifenprodil, the β-amino­alcohol substructure of ifenprodil was incorporated into a ring system resulting in seven-membered 3-benzazepines with high GluN2B affinity, high selectivity over related receptors and high metabolic stability (Tewes *et al.*, 2010*a* ▸,**b* ▸;* Schepmann *et al.*, 2010 ▸; Falck *et al.*, 2014 ▸).

## Elucidation of the relative configuration   

**Figure 1:**
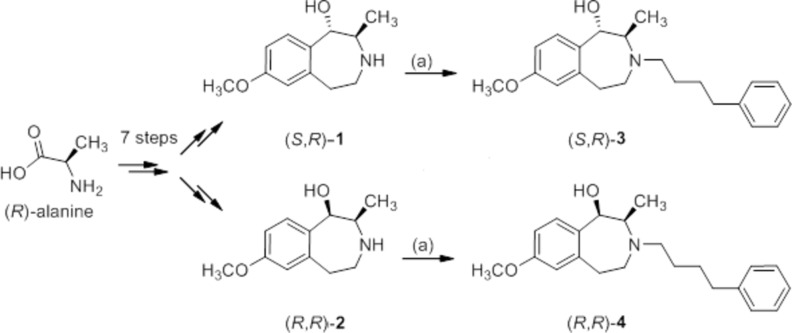
Reaction scheme. Reagents and reaction conditions: (*a*) 1-chloro-4-phenyl­butane, CH_3_CN, Bu_4_NI, K_2_CO_3_, Δ, 72 h.

The 3-benzazepines (**3**) and (**4**) were prepared in a chiral pool synthesis starting with (*R*)-alanine. In a seven-step sequence the secondary amines (*S*,*R*)-**1** and (*R*,*R*)-**2** were obtained. In the last step, the secondary amines (*S*,*R*)-**1** and (*R*,*R*)-**2** were alkyl­ated with 1-chloro-4-phenyl­butane to afford the conformationally constrained ifenprodil analogues (**3**) and (**4**) which reveal high GluN2B affinity with *K*_i_ values of 47 n*M* and 41 n*M*, respectively (Tewes *et al.*, 2015 ▸) (Fig. 1 ▸).
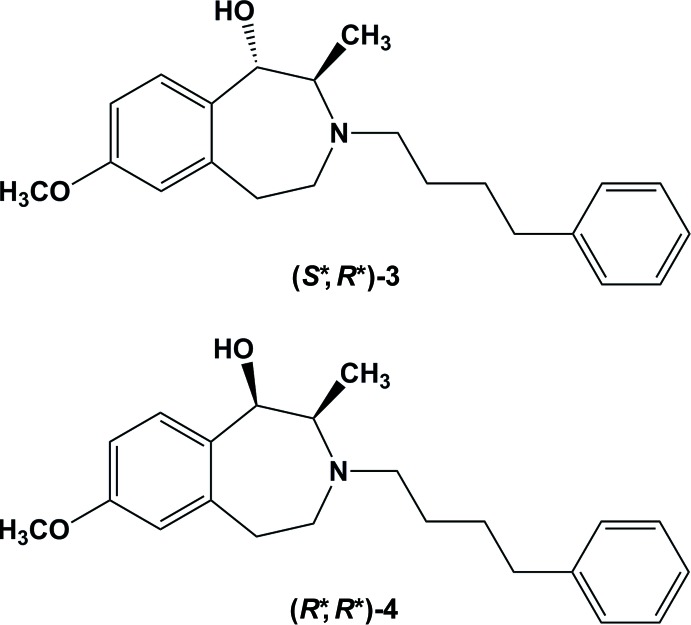


As a result of the flexibility of the tetra­hydro-3-benzazepine system of (**1**)–(**4**), the relative configuration of the 3-benz­azepines (**3**) and (**4**) could not be determined unequivocally by inter­pretation of NMR spectra. However, crystallization of 70:30 mixtures of (*S*,*R*)-**3** and (*R*,*S*)-**3**, as well as (*R*,*R*)-**4** and (*S*,*S*)-**4**, led to colourless crystals which were suitable for X-ray crystal structure analysis. In both cases, the crystals proved to be of a racemic mixture, with the compounds having relative configurations (S*,R*)-**3** and (R*,R*)-**4**.

## Structural commentary   

**Figure 2:**
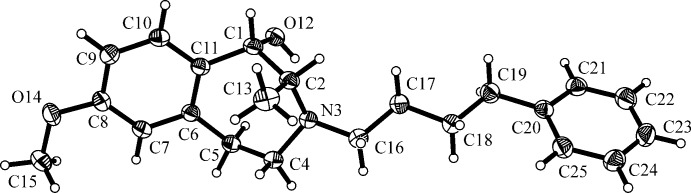
The mol­ecular structure of compound (S*,R*)-**3**, with atom labelling. Displacement ellipsoids are drawn at the 30% probability level.

**Figure 3:**
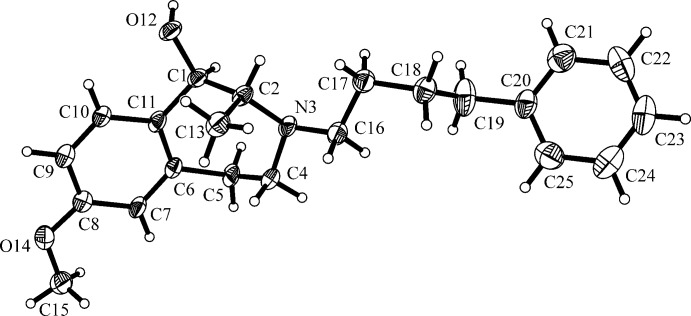
The mol­ecular structure of compound (R*,R*)-**4**, with atom labelling. Displacement ellipsoids are drawn at the 30% probability level.

The mol­ecular structures of compounds (S*,R*)-**3** and (R*,R*)-**4** are depicted in Figs. 2 ▸ and 3 ▸, respectively. In the structure of (S*,R*)-**3** (Fig. 2 ▸), a *trans*-configuration with *−anti-periplanar* conformation and a torsion angle O12—C1—C2—C13 = −175.00 (12)°, of the OH group and the methyl group at the seven-membered azepine ring is shown. In (R*,R*)-**4** (Fig. 3 ▸) the same substituents are *cis*-configured, in *+syn-clinal* conformation with torsion angle O12—C1—C2—C13 = 73.2 (7)°.

In compound (S*,R*)-**3** the 4-phenyl­butyl side chain adopts an extended conformation [torsion angle C16—C17—C18—C19 = 172.13 (14)°]. The CH_3_ and OH groups are on opposite sides of the azepine ring adopting an almost axial orientation. The bonds between atom N3 and its adjacent C atoms (C2, C16, C4) are shorter (ca. 1.47 Å) than the C—C bonds in the azepine ring (*ca* 1.52–1.54 Å). There is an intra­molecular O-H⋯N contact present (Table 1 ▸) involving the O12 hydroxyl group and atom N3 of the 3-benzazepine ring, enclosing an *S*(5) ring motif.

In compound (R*,R*)-**4** the 4-phenyl­butyl side chain exists in a twisted conformation torsion angle C16—C17—C18—C19 = 76.1 (9)°]. The CH_3_ group is on the opposite side of the azepine ring adopting an almost axial orientation, as for (S*,R*)-**3**. However, here the OH group adopts a more equatorial orientation at the seven-membered azepine ring, in contrast to the OH group of (S*,R*)-**3**. The angles of the aliphatic part of the 3-benzazepine ring are close to the tetra­hedral angle value.

## Supra­molecular features   

**Figure 4:**
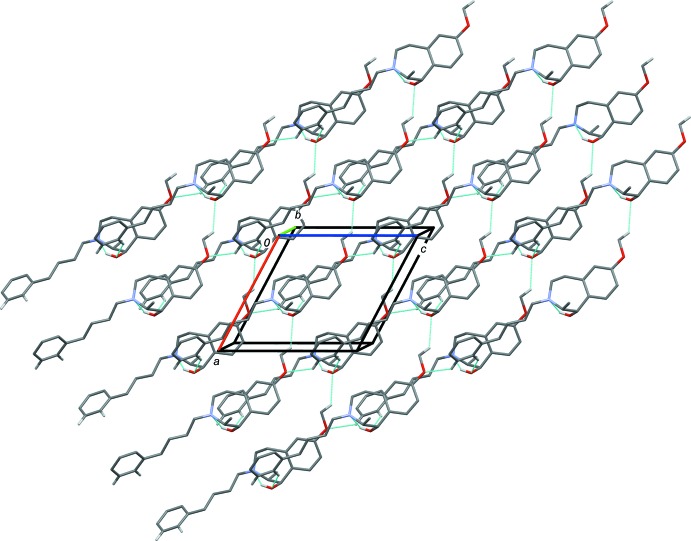
A view along the *b* axis of the crystal packing of compound (S*,R*)-**3**. The hydrogen bonds are shown as dashed lines (see Table 1 ▸); for clarity, only the H atoms involved in these inter­actions are included.

**Figure 5:**
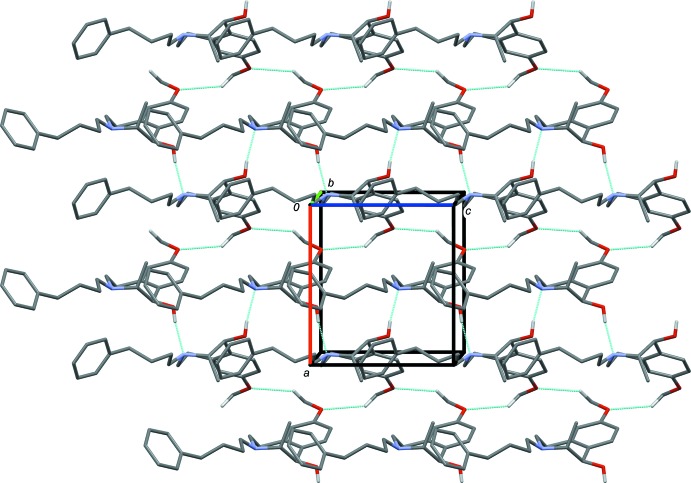
A view along the *b* axis of the crystal packing of compound (R*,R*)-**4**. The hydrogen bonds are shown as dashed lines (see Table 2 ▸; for clarity, only the H atoms involved in these inter­actions are included.

In the crystal of (S*,R*)-**3**, mol­ecules are linked *via* C—H⋯O hydrogen bonds, forming slabs parallel to the *ac* plane (Table 1 ▸ and Fig. 4 ▸). In the crystal of (R*,R*)-**4**, mol­ecules, are linked *via* O—H⋯N hydrogen bonds, forming chains propagating along the *c-*axis direction. The chains are linked by C—H⋯O hydrogen bonds, forming slabs parallel to the *ac* plane (Table 2 ▸ and Fig. 5 ▸).

## Synthesis and crystallization   

**(1** S* **,2** R* **)-7-Meth­oxy-2-methyl-3-(4-phenyl­but­yl)-2,3,4,5-tetra­hydro-1** ***H*** **-3-benzazepin-1-ol: (** ***S*** *,***R*** *)-3

As described for the synthesis of (*R*,*S*)-**3** (Tewes *et al.*, 2015 ▸), the enanti­omer (*S*,*R*)-**3** was prepared in the same manner by alkyl­ation of secondary amine (*S*,*R*)-**1** [(*S*,*R*)-**1**:(*R*,*S*)-**1** = 70:30] with 1-chloro-4-phenyl­butane. Purification by flash chromatography (2 cm, *n*-hexa­ne:ethyl acetate 95:5 and 1% *N,N*-di­methyl­ethanamine, 10 ml, *R*_f_ = 0.10) resulted in colourless crystals. The sample, contained the enanti­omers (*S*,*R*)-**3** and (*R*,*S*)-**3** in the ratio 70:30. Spectroscopic data are given in Tewes *et al.* (2015 ▸).

**(1** R* **,2** R* **)-7-Meth­oxy-2-methyl-3-(4-phenyl­but­yl)-2,3,4,5-tetra­hydro-1** ***H*** **-3-benzazepin-1-ol: (** ***R*** *,***R*** *)-4

As described for the synthesis of (*S*,*S*)-**4** (Tewes *et al.*, 2015 ▸), the enanti­omer (*R*,*R*)-**4** was prepared in the same manner by alkyl­ation of secondary amine (*R*,*R*)-**2** [(*R*,*R*)-**1**:(*S*,*S*)-**1** = 70:30] with 1-chloro-4-phenyl­butane. Purification by flash chromatography (2 cm, *n*-hexa­ne:ethyl acetate 70: 30 and 1% *N,N*-di­methyl­ethanamine, 10 ml, *R*_f_ = 0.29) resulted in colourless crystals. The sample contained the enanti­omers (*R*,*R*)-**4** and (*S*,*S*)-**4** in the ratio 70:30. Spectroscopic data are given in Tewes *et al.* (2015 ▸).

In both cases, the compounds were used for recrystallization with ethyl acetate and the crystals obtained were used for the subsequent X-ray crystal structure analyses. The crystals thus obtained proved to be racemic mixtures, with the compounds having relative configurations (R*,S*)-**3** and (R*,R*)-**4**.

## Refinement details   

Crystal data, data collection and structure refinement details are summarized in Table 3 ▸. For both compounds the OH and C-bound H atoms were included in calculated positions and treated as riding atoms: O—H = 0.83 Å, C—H = 0.94–0.99 Å with *U*_iso_(H) = 1.5*U*_eq_(O or C-meth­yl) and 1.2*U*_eq_(C) for other H atoms.

## Supplementary Material

Crystal structure: contains datablock(s) SR-3, RR-4, global. DOI: 10.1107/S2056989016005843/su5285sup1.cif

Structure factors: contains datablock(s) S,R-3. DOI: 10.1107/S2056989016005843/su5285SR-3sup3.hkl

Structure factors: contains datablock(s) R,R-4. DOI: 10.1107/S2056989016005843/su5285RR-4sup2.hkl

Supporting information file. DOI: 10.1107/S2056989016005843/su5285SR-3sup4.cml

Supporting information file. DOI: 10.1107/S2056989016005843/su5285RR-4sup5.cml

CCDC references: 1472946, 1472945

Additional supporting information:  crystallographic information; 3D view; checkCIF report

## Figures and Tables

**Table 1:** Hydrogen-bond geometry (Å, °) for (R*,S*)-**3** [Chem scheme1]

*D*—H⋯*A*	*D*—H	H⋯*A*	*D*⋯*A*	*D*—H⋯*A*
O12—H12⋯N3	0.83	2.17	2.6883 (17)	120
C15—H15*B*⋯O12^i^	0.97	2.59	3.295 (2)	130
C21—H21⋯O12^ii^	0.94	2.55	3.349 (2)	143
C22—H22⋯O14^iii^	0.94	2.59	3.373 (3)	141

Symmetry codes: (i) 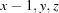; (ii) 
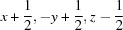
; (iii) 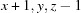.

**Table 2:** Hydrogen-bond geometry (Å, °) for (R*,R*)-**4** [Chem scheme1]

*D*—H⋯*A*	*D*—H	H⋯*A*	*D*⋯*A*	*D*—H⋯*A*
O12—H12⋯N3^i^	0.83	1.97	2.796 (6)	172
C15—H15*C*⋯O14^ii^	0.97	2.58	3.365 (9)	138

Symmetry codes: (i) 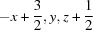; (ii) 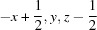.

**Table 3:** Experimental details

	(R*,S*)-**3**	(R*,R*)-**4**
Crystal data		
Chemical formula	C_22_H_29_NO_2_	C_22_H_29_NO_2_
*M*_r_	339.46	339.46
Crystal system, space group	Monoclinic, *P*2_1_/*n*	Orthorhombic, *P* *c* *a*2_1_
Temperature (K)	223	223
*a*, *b*, *c* (Å)	10.3594 (2), 18.8246 (4), 10.9981 (3)	9.2049 (5), 25.4468 (17), 8.2451 (6)
α, β, γ (°)	90, 117.889 (1), 90	90, 90, 90
*V* (Å^3^)	1895.65 (8)	1931.3 (2)
*Z*	4	4
Radiation type	Cu *K*α	Cu *K*α
μ (mm^−1^)	0.59	0.58
Crystal size (mm)	0.40 × 0.25 × 0.10	0.35 × 0.05 × 0.03

Data collection		
Diffractometer	Nonius KappaCCD APEXII	Nonius KappaCCD APEXII
Absorption correction	Multi-scan (*DENZO*; Otwinowski *et al.*, 2003 ▸)	Multi-scan (*DENZO*; Otwinowski *et al.*, 2003 ▸)
*T*_min_, *T*_max_	0.799, 0.944	0.824, 0.983
No. of measured, independent and observed [*I* > 2σ(*I*)] reflections	8812, 3077, 2864	8312, 2885, 2164
*R*_int_	0.034	0.082
(sin θ/λ)_max_ (Å^−1^)	0.600	0.599

Refinement		
*R*[*F* ^2^ > 2σ(*F* ^2^)], *wR*(*F* ^2^), *S*	0.045, 0.118, 1.04	0.087, 0.231, 1.25
No. of reflections	3077	2885
No. of parameters	229	229
No. of restraints	0	1
H-atom treatment	H-atom parameters constrained	H-atom parameters constrained
Δρ_max_, Δρ_min_ (e Å^−3^)	0.16, −0.13	0.26, −0.25

Computer programs: *COLLECT* (Nonius, 1998 ▸), *DENZO–SMN* (Otwinowski & Minor, 1997 ▸), *SHELXS97*, *SHELXL97* and *XP* in *SHELXTL* (Sheldrick, 2008 ▸), *Mercury* (Macrae *et al.*, 2008 ▸) and *PLATON* (Spek, 2009 ▸).

## supplementary crystallographic information

### (SR-3) (1S*,2R*)-7-Methoxy-2-methyl-3-(4-phenylbutyl)-2,3,4,5-tetrahydro-1H-3-benzazepin-1-ol Crystal data

**Table:**

C_22_H_29_NO_2_	*F*(000) = 736
*M**_r_* = 339.46	*D*_x_ = 1.189 Mg m^−^^3^
Monoclinic, *P*2_1_/*n*	Cu *K*α radiation, λ = 1.54178 Å
Hall symbol: -P 2yn	Cell parameters from 1877 reflections
*a* = 10.3594 (2) Å	θ = 0.9–68.3°
*b* = 18.8246 (4) Å	µ = 0.59 mm^−^^1^
*c* = 10.9981 (3) Å	*T* = 223 K
β = 117.889 (1)°	Plate, colourless
*V* = 1895.65 (8) Å^3^	0.40 × 0.25 × 0.10 mm
*Z* = 4	

### (SR-3) (1S*,2R*)-7-Methoxy-2-methyl-3-(4-phenylbutyl)-2,3,4,5-tetrahydro-1H-3-benzazepin-1-ol Data collection

**Table:**

Nonius KappaCCD APEXII diffractometer	3077 independent reflections
Radiation source: fine-focus sealed tube	2864 reflections with *I* > 2σ(*I*)
Graphite monochromator	*R*_int_ = 0.034
ω and \ scans	θ_max_ = 67.6°, θ_min_ = 5.1°
Absorption correction: multi-scan (*DENZO*; Otwinowski *et al.*, 2003)	*h* = 0→12
*T*_min_ = 0.799, *T*_max_ = 0.944	*k* = 0→21
8812 measured reflections	*l* = −13→11

### (SR-3) (1S*,2R*)-7-Methoxy-2-methyl-3-(4-phenylbutyl)-2,3,4,5-tetrahydro-1H-3-benzazepin-1-ol Refinement

**Table:**

Refinement on *F*^2^	Primary atom site location: structure-invariant direct methods
Least-squares matrix: full	Secondary atom site location: difference Fourier map
*R*[*F*^2^ > 2σ(*F*^2^)] = 0.045	Hydrogen site location: inferred from neighbouring sites
*wR*(*F*^2^) = 0.118	H-atom parameters constrained
*S* = 1.04	*w* = 1/[σ^2^(*F*_o_^2^) + (0.056*P*)^2^ + 0.5327*P*] where *P* = (*F*_o_^2^ + 2*F*_c_^2^)/3
3077 reflections	(Δ/σ)_max_ < 0.001
229 parameters	Δρ_max_ = 0.16 e Å^−^^3^
0 restraints	Δρ_min_ = −0.13 e Å^−^^3^

### (SR-3) (1S*,2R*)-7-Methoxy-2-methyl-3-(4-phenylbutyl)-2,3,4,5-tetrahydro-1H-3-benzazepin-1-ol Special details

**Table:**

Geometry. All esds (except the esd in the dihedral angle between two l.s. planes) are estimated using the full covariance matrix. The cell esds are taken into account individually in the estimation of esds in distances, angles and torsion angles; correlations between esds in cell parameters are only used when they are defined by crystal symmetry. An approximate (isotropic) treatment of cell esds is used for estimating esds involving l.s. planes.
Refinement. Refinement of F^2^ against ALL reflections. The weighted R-factor wR and goodness of fit S are based on F^2^, conventional R-factors R are based on F, with F set to zero for negative F^2^. The threshold expression of F^2^ > 2sigma(F^2^) is used only for calculating R-factors(gt) etc. and is not relevant to the choice of reflections for refinement. R-factors based on F^2^ are statistically about twice as large as those based on F, and R- factors based on ALL data will be even larger.

### (SR-3) (1S*,2R*)-7-Methoxy-2-methyl-3-(4-phenylbutyl)-2,3,4,5-tetrahydro-1H-3-benzazepin-1-ol Fractional atomic coordinates and isotropic or equivalent isotropic displacement parameters (Å^2^)

**Table:**

	*x*	*y*	*z*	*U*_iso_*/*U*_eq_	
C1	0.66460 (15)	0.15839 (8)	0.42463 (15)	0.0438 (4)	
H1	0.7477	0.1434	0.5128	0.053*	
C2	0.64344 (16)	0.10045 (8)	0.31824 (16)	0.0461 (4)	
H2	0.7422	0.0894	0.3305	0.055*	
N3	0.56166 (13)	0.13021 (7)	0.17856 (13)	0.0450 (3)	
C4	0.40433 (16)	0.13908 (9)	0.12903 (16)	0.0489 (4)	
H4A	0.3643	0.0943	0.1422	0.059*	
H4B	0.3575	0.1489	0.0301	0.059*	
C5	0.36506 (16)	0.19818 (9)	0.20015 (16)	0.0470 (4)	
H5A	0.4188	0.2409	0.1996	0.056*	
H5B	0.2608	0.2086	0.1447	0.056*	
C6	0.39431 (15)	0.18510 (7)	0.34623 (15)	0.0417 (3)	
C7	0.27908 (16)	0.19112 (8)	0.37801 (16)	0.0448 (4)	
H7	0.1850	0.2020	0.3078	0.054*	
C8	0.30111 (17)	0.18140 (8)	0.51121 (17)	0.0456 (4)	
C9	0.43943 (18)	0.16572 (9)	0.61484 (17)	0.0500 (4)	
H9	0.4556	0.1595	0.7057	0.060*	
C10	0.55380 (17)	0.15931 (8)	0.58400 (16)	0.0475 (4)	
H10	0.6474	0.1483	0.6549	0.057*	
C11	0.53452 (15)	0.16869 (7)	0.45093 (15)	0.0421 (3)	
O12	0.70726 (11)	0.22260 (6)	0.38429 (11)	0.0492 (3)	
H12	0.6794	0.2218	0.3002	0.074*	
C13	0.5846 (2)	0.03100 (9)	0.3454 (2)	0.0621 (5)	
H13A	0.4891	0.0393	0.3389	0.093*	
H13B	0.6509	0.0138	0.4368	0.093*	
H13C	0.5763	−0.0042	0.2777	0.093*	
O14	0.19492 (12)	0.18687 (7)	0.55197 (13)	0.0607 (3)	
C15	0.04982 (18)	0.20161 (11)	0.4508 (2)	0.0653 (5)	
H15A	0.0473	0.2467	0.4069	0.098*	
H15B	−0.0131	0.2041	0.4939	0.098*	
H15C	0.0159	0.1642	0.3822	0.098*	
C16	0.59495 (18)	0.09299 (9)	0.07866 (17)	0.0528 (4)	
H16A	0.5150	0.1010	−0.0142	0.063*	
H16B	0.6004	0.0418	0.0970	0.063*	
C17	0.73646 (17)	0.11704 (9)	0.08386 (17)	0.0497 (4)	
H17A	0.8169	0.1068	0.1754	0.060*	
H17B	0.7329	0.1686	0.0703	0.060*	
C18	0.76778 (17)	0.08148 (9)	−0.02367 (17)	0.0492 (4)	
H18A	0.7855	0.0307	−0.0025	0.059*	
H18B	0.6819	0.0861	−0.1141	0.059*	
C19	0.89835 (18)	0.11362 (8)	−0.02919 (18)	0.0511 (4)	
H19A	0.9830	0.1083	0.0619	0.061*	
H19B	0.8804	0.1647	−0.0464	0.061*	
C20	0.93912 (16)	0.08411 (8)	−0.13445 (15)	0.0439 (4)	
C21	1.04410 (18)	0.11928 (9)	−0.15571 (17)	0.0521 (4)	
H21	1.0858	0.1611	−0.1062	0.063*	
C22	1.0888 (2)	0.09450 (12)	−0.24730 (19)	0.0661 (5)	
H22	1.1605	0.1193	−0.2596	0.079*	
C23	1.0292 (2)	0.03362 (12)	−0.32099 (19)	0.0683 (5)	
H23	1.0608	0.0162	−0.3826	0.082*	
C24	0.9229 (2)	−0.00145 (10)	−0.30370 (19)	0.0638 (5)	
H24	0.8802	−0.0426	−0.3554	0.077*	
C25	0.87799 (18)	0.02311 (8)	−0.21089 (18)	0.0533 (4)	
H25	0.8056	−0.0017	−0.1996	0.064*	

### (SR-3) (1S*,2R*)-7-Methoxy-2-methyl-3-(4-phenylbutyl)-2,3,4,5-tetrahydro-1H-3-benzazepin-1-ol Atomic displacement parameters (Å^2^)

**Table:**

	*U*^11^	*U*^22^	*U*^33^	*U*^12^	*U*^13^	*U*^23^
C1	0.0332 (7)	0.0498 (8)	0.0415 (8)	0.0023 (6)	0.0116 (7)	−0.0005 (6)
C2	0.0356 (7)	0.0512 (8)	0.0452 (9)	0.0044 (6)	0.0135 (7)	−0.0032 (6)
N3	0.0365 (6)	0.0552 (7)	0.0404 (7)	0.0007 (5)	0.0155 (6)	−0.0060 (5)
C4	0.0364 (8)	0.0644 (10)	0.0401 (8)	0.0007 (6)	0.0130 (7)	−0.0025 (7)
C5	0.0350 (7)	0.0598 (9)	0.0402 (9)	0.0078 (6)	0.0127 (7)	0.0047 (6)
C6	0.0362 (7)	0.0444 (7)	0.0416 (8)	0.0008 (6)	0.0157 (7)	−0.0010 (6)
C7	0.0342 (7)	0.0486 (8)	0.0465 (9)	0.0010 (6)	0.0145 (7)	0.0003 (6)
C8	0.0427 (8)	0.0463 (8)	0.0524 (9)	−0.0032 (6)	0.0259 (8)	0.0006 (6)
C9	0.0482 (9)	0.0588 (9)	0.0422 (9)	0.0015 (7)	0.0205 (8)	0.0050 (7)
C10	0.0393 (8)	0.0554 (9)	0.0404 (8)	0.0039 (6)	0.0125 (7)	0.0047 (6)
C11	0.0362 (7)	0.0444 (8)	0.0413 (8)	0.0005 (6)	0.0145 (7)	−0.0007 (6)
O12	0.0407 (6)	0.0532 (6)	0.0529 (7)	−0.0060 (4)	0.0212 (6)	−0.0065 (5)
C13	0.0674 (11)	0.0498 (9)	0.0614 (11)	0.0020 (8)	0.0236 (10)	0.0011 (8)
O14	0.0451 (6)	0.0837 (8)	0.0618 (8)	0.0015 (5)	0.0321 (6)	0.0090 (6)
C15	0.0398 (9)	0.0861 (13)	0.0710 (13)	−0.0001 (8)	0.0267 (9)	−0.0064 (9)
C16	0.0457 (9)	0.0631 (10)	0.0487 (9)	−0.0033 (7)	0.0213 (8)	−0.0118 (7)
C17	0.0466 (9)	0.0521 (9)	0.0500 (9)	0.0022 (6)	0.0225 (8)	−0.0040 (7)
C18	0.0442 (8)	0.0551 (9)	0.0473 (9)	0.0006 (6)	0.0206 (8)	−0.0021 (7)
C19	0.0508 (9)	0.0485 (8)	0.0558 (10)	−0.0009 (7)	0.0264 (8)	−0.0040 (7)
C20	0.0388 (8)	0.0473 (8)	0.0413 (8)	0.0073 (6)	0.0152 (7)	0.0063 (6)
C21	0.0472 (9)	0.0595 (9)	0.0452 (9)	−0.0030 (7)	0.0179 (8)	0.0050 (7)
C22	0.0559 (10)	0.0947 (14)	0.0531 (11)	−0.0001 (9)	0.0299 (9)	0.0124 (10)
C23	0.0643 (11)	0.0977 (15)	0.0456 (10)	0.0195 (10)	0.0281 (9)	0.0025 (9)
C24	0.0610 (11)	0.0631 (11)	0.0583 (11)	0.0095 (8)	0.0205 (9)	−0.0109 (8)
C25	0.0485 (9)	0.0517 (9)	0.0611 (10)	0.0016 (7)	0.0267 (8)	−0.0014 (7)

### (SR-3) (1S*,2R*)-7-Methoxy-2-methyl-3-(4-phenylbutyl)-2,3,4,5-tetrahydro-1H-3-benzazepin-1-ol Geometric parameters (Å, º)

**Table:**

C1—O12	1.4271 (18)	C9—C10	1.382 (2)
C1—C11	1.517 (2)	C10—C11	1.393 (2)
C1—C2	1.539 (2)	O14—C15	1.418 (2)
C2—N3	1.474 (2)	C16—C17	1.510 (2)
C2—C13	1.530 (2)	C17—C18	1.520 (2)
N3—C4	1.4664 (19)	C18—C19	1.509 (2)
N3—C16	1.4736 (19)	C19—C20	1.511 (2)
C4—C5	1.521 (2)	C20—C21	1.383 (2)
C5—C6	1.509 (2)	C20—C25	1.389 (2)
C6—C7	1.397 (2)	C21—C22	1.372 (3)
C6—C11	1.402 (2)	C22—C23	1.372 (3)
C7—C8	1.385 (2)	C23—C24	1.371 (3)
C8—O14	1.3721 (18)	C24—C25	1.385 (2)
C8—C9	1.383 (2)		
			
O12—C1—C11	112.51 (12)	C10—C9—C8	119.56 (14)
O12—C1—C2	108.58 (12)	C9—C10—C11	121.94 (14)
C11—C1—C2	114.35 (12)	C10—C11—C6	118.37 (13)
N3—C2—C13	116.16 (13)	C10—C11—C1	118.72 (13)
N3—C2—C1	109.33 (12)	C6—C11—C1	122.88 (13)
C13—C2—C1	112.66 (13)	C8—O14—C15	118.41 (13)
C4—N3—C16	112.60 (12)	N3—C16—C17	113.02 (13)
C4—N3—C2	115.30 (12)	C16—C17—C18	113.29 (13)
C16—N3—C2	112.04 (12)	C19—C18—C17	112.11 (13)
N3—C4—C5	114.23 (13)	C18—C19—C20	117.42 (14)
C6—C5—C4	117.34 (13)	C21—C20—C25	117.73 (15)
C7—C6—C11	119.38 (14)	C21—C20—C19	118.49 (14)
C7—C6—C5	118.92 (13)	C25—C20—C19	123.78 (14)
C11—C6—C5	121.68 (13)	C22—C21—C20	121.55 (17)
C8—C7—C6	121.11 (14)	C21—C22—C23	120.28 (17)
O14—C8—C9	115.27 (14)	C24—C23—C22	119.27 (17)
O14—C8—C7	125.09 (14)	C23—C24—C25	120.67 (18)
C9—C8—C7	119.63 (14)	C24—C25—C20	120.47 (16)
			
O12—C1—C2—N3	−44.25 (15)	C7—C6—C11—C1	177.61 (13)
C11—C1—C2—N3	82.30 (15)	C5—C6—C11—C1	−3.9 (2)
O12—C1—C2—C13	−175.00 (12)	O12—C1—C11—C10	−114.59 (15)
C11—C1—C2—C13	−48.45 (18)	C2—C1—C11—C10	120.93 (15)
C13—C2—N3—C4	52.51 (18)	O12—C1—C11—C6	67.47 (18)
C1—C2—N3—C4	−76.33 (15)	C2—C1—C11—C6	−57.01 (19)
C13—C2—N3—C16	−78.04 (16)	C9—C8—O14—C15	−178.90 (15)
C1—C2—N3—C16	153.12 (12)	C7—C8—O14—C15	1.9 (2)
C16—N3—C4—C5	−158.97 (13)	C4—N3—C16—C17	147.99 (14)
C2—N3—C4—C5	70.75 (17)	C2—N3—C16—C17	−80.09 (17)
N3—C4—C5—C6	−72.96 (18)	N3—C16—C17—C18	−177.17 (14)
C4—C5—C6—C7	−123.71 (15)	C16—C17—C18—C19	172.13 (14)
C4—C5—C6—C11	57.74 (19)	C17—C18—C19—C20	−178.61 (13)
C11—C6—C7—C8	0.2 (2)	C18—C19—C20—C21	170.56 (14)
C5—C6—C7—C8	−178.36 (13)	C18—C19—C20—C25	−10.0 (2)
C6—C7—C8—O14	179.40 (14)	C25—C20—C21—C22	−1.1 (2)
C6—C7—C8—C9	0.2 (2)	C19—C20—C21—C22	178.45 (15)
O14—C8—C9—C10	−179.82 (14)	C20—C21—C22—C23	0.2 (3)
C7—C8—C9—C10	−0.6 (2)	C21—C22—C23—C24	1.0 (3)
C8—C9—C10—C11	0.5 (2)	C22—C23—C24—C25	−1.4 (3)
C9—C10—C11—C6	0.0 (2)	C23—C24—C25—C20	0.5 (3)
C9—C10—C11—C1	−178.04 (14)	C21—C20—C25—C24	0.7 (2)
C7—C6—C11—C10	−0.3 (2)	C19—C20—C25—C24	−178.77 (16)
C5—C6—C11—C10	178.20 (14)		

### (SR-3) (1S*,2R*)-7-Methoxy-2-methyl-3-(4-phenylbutyl)-2,3,4,5-tetrahydro-1H-3-benzazepin-1-ol Hydrogen-bond geometry (Å, º)

**Table:**

*D*—H···*A*	*D*—H	H···*A*	*D*···*A*	*D*—H···*A*
O12—H12···N3	0.83	2.17	2.6883 (17)	120
C15—H15*B*···O12^i^	0.97	2.59	3.295 (2)	130
C21—H21···O12^ii^	0.94	2.55	3.349 (2)	143
C22—H22···O14^iii^	0.94	2.59	3.373 (3)	141

Symmetry codes: (i) *x*−1, *y*, *z*; (ii) *x*+1/2, −*y*+1/2, *z*−1/2; (iii) *x*+1, *y*, *z*−1.

### (RR-4) (1R*,2R*)-7-Methoxy-2-methyl-3-(4-phenylbutyl)-2,3,4,5-tetrahydro-1H-3-benzazepin-1-ol Crystal data

**Table:**

C_22_H_29_NO_2_	*F*(000) = 736
*M**_r_* = 339.46	*D*_x_ = 1.167 Mg m^−^^3^
Orthorhombic, *P**c**a*2_1_	Cu *K*α radiation, λ = 1.54178 Å
Hall symbol: P 2c -2ac	Cell parameters from 2216 reflections
*a* = 9.2049 (5) Å	θ = 0.9–70.1°
*b* = 25.4468 (17) Å	µ = 0.57 mm^−^^1^
*c* = 8.2451 (6) Å	*T* = 223 K
*V* = 1931.3 (2) Å^3^	Needle, colourless
*Z* = 4	0.35 × 0.05 × 0.03 mm

### (RR-4) (1R*,2R*)-7-Methoxy-2-methyl-3-(4-phenylbutyl)-2,3,4,5-tetrahydro-1H-3-benzazepin-1-ol Data collection

**Table:**

Nonius KappaCCD APEXII diffractometer	2885 independent reflections
Radiation source: fine-focus sealed tube	2164 reflections with *I* > 2σ(*I*)
Graphite monochromator	*R*_int_ = 0.082
ω and φ scans	θ_max_ = 67.5°, θ_min_ = 3.5°
Absorption correction: multi-scan (*DENZO*; Otwinowski *et al.*, 2003)	*h* = −10→10
*T*_min_ = 0.824, *T*_max_ = 0.983	*k* = −30→30
8312 measured reflections	*l* = −9→9

### (RR-4) (1R*,2R*)-7-Methoxy-2-methyl-3-(4-phenylbutyl)-2,3,4,5-tetrahydro-1H-3-benzazepin-1-ol Refinement

**Table:**

Refinement on *F*^2^	Primary atom site location: structure-invariant direct methods
Least-squares matrix: full	Secondary atom site location: difference Fourier map
*R*[*F*^2^ > 2σ(*F*^2^)] = 0.087	Hydrogen site location: inferred from neighbouring sites
*wR*(*F*^2^) = 0.231	H-atom parameters constrained
*S* = 1.25	*w* = 1/[σ^2^(*F*_o_^2^) + (0.0636*P*)^2^ + 2.9552*P*] where *P* = (*F*_o_^2^ + 2*F*_c_^2^)/3
2885 reflections	(Δ/σ)_max_ < 0.001
229 parameters	Δρ_max_ = 0.26 e Å^−^^3^
1 restraint	Δρ_min_ = −0.24 e Å^−^^3^

### (RR-4) (1R*,2R*)-7-Methoxy-2-methyl-3-(4-phenylbutyl)-2,3,4,5-tetrahydro-1H-3-benzazepin-1-ol Special details

**Table:**

Geometry. All esds (except the esd in the dihedral angle between two l.s. planes) are estimated using the full covariance matrix. The cell esds are taken into account individually in the estimation of esds in distances, angles and torsion angles; correlations between esds in cell parameters are only used when they are defined by crystal symmetry. An approximate (isotropic) treatment of cell esds is used for estimating esds involving l.s. planes.
Refinement. Refinement of F^2^ against ALL reflections. The weighted R-factor wR and goodness of fit S are based on F^2^, conventional R-factors R are based on F, with F set to zero for negative F^2^. The threshold expression of F^2^ > 2sigma(F^2^) is used only for calculating R-factors(gt) etc. and is not relevant to the choice of reflections for refinement. R-factors based on F^2^ are statistically about twice as large as those based on F, and R- factors based on ALL data will be even larger.

### (RR-4) (1R*,2R*)-7-Methoxy-2-methyl-3-(4-phenylbutyl)-2,3,4,5-tetrahydro-1H-3-benzazepin-1-ol Fractional atomic coordinates and isotropic or equivalent isotropic displacement parameters (Å^2^)

**Table:**

	*x*	*y*	*z*	*U*_iso_*/*U*_eq_	
C1	0.6240 (7)	0.2962 (3)	−0.1209 (7)	0.0427 (15)	
H1	0.7154	0.3049	−0.1780	0.051*	
C2	0.5358 (6)	0.2577 (3)	−0.2273 (7)	0.0415 (16)	
H2	0.5846	0.2233	−0.2157	0.050*	
N3	0.5442 (5)	0.2708 (2)	−0.4043 (6)	0.0447 (13)	
C4	0.4711 (8)	0.3205 (3)	−0.4472 (7)	0.0514 (18)	
H4A	0.3709	0.3191	−0.4074	0.062*	
H4B	0.4670	0.3233	−0.5656	0.062*	
C5	0.5428 (8)	0.3701 (3)	−0.3805 (7)	0.0524 (18)	
H5A	0.5133	0.4002	−0.4472	0.063*	
H5B	0.6484	0.3665	−0.3898	0.063*	
C6	0.5040 (7)	0.3814 (3)	−0.2034 (7)	0.0416 (16)	
C7	0.4270 (7)	0.4272 (3)	−0.1680 (7)	0.0471 (16)	
H7	0.3990	0.4500	−0.2520	0.057*	
C8	0.3912 (8)	0.4391 (3)	−0.0060 (8)	0.0554 (18)	
C9	0.4298 (8)	0.4036 (3)	0.1137 (8)	0.062 (2)	
H9	0.4046	0.4105	0.2221	0.075*	
C10	0.5042 (8)	0.3586 (3)	0.0767 (7)	0.0537 (19)	
H10	0.5286	0.3352	0.1605	0.064*	
C11	0.5447 (6)	0.3465 (3)	−0.0823 (7)	0.0427 (16)	
O12	0.6590 (5)	0.26820 (19)	0.0243 (5)	0.0512 (12)	
H12	0.7485	0.2676	0.0365	0.077*	
C13	0.3828 (7)	0.2497 (3)	−0.1623 (9)	0.0564 (18)	
H13A	0.3254	0.2810	−0.1823	0.085*	
H13B	0.3870	0.2430	−0.0466	0.085*	
H13C	0.3383	0.2199	−0.2165	0.085*	
O14	0.3170 (6)	0.4827 (2)	0.0418 (6)	0.0726 (16)	
C15	0.2708 (10)	0.5186 (3)	−0.0778 (9)	0.071 (2)	
H15A	0.3543	0.5312	−0.1380	0.106*	
H15B	0.2221	0.5480	−0.0264	0.106*	
H15C	0.2040	0.5012	−0.1514	0.106*	
C16	0.4847 (7)	0.2273 (3)	−0.5048 (9)	0.0536 (18)	
H16A	0.4947	0.2372	−0.6192	0.064*	
H16B	0.3806	0.2242	−0.4819	0.064*	
C17	0.5525 (7)	0.1746 (3)	−0.4821 (8)	0.0526 (17)	
H17A	0.5222	0.1603	−0.3771	0.063*	
H17B	0.6584	0.1785	−0.4801	0.063*	
C18	0.5110 (8)	0.1357 (3)	−0.6165 (8)	0.0556 (18)	
H18A	0.5324	0.0998	−0.5800	0.067*	
H18B	0.4063	0.1380	−0.6369	0.067*	
C19	0.5930 (12)	0.1465 (4)	−0.7735 (10)	0.090 (3)	
H19A	0.5682	0.1819	−0.8114	0.108*	
H19B	0.6975	0.1459	−0.7510	0.108*	
C20	0.5606 (9)	0.1078 (3)	−0.9069 (9)	0.062 (2)	
C21	0.6546 (10)	0.0668 (4)	−0.9347 (10)	0.077 (2)	
H21	0.7380	0.0627	−0.8702	0.092*	
C22	0.6251 (13)	0.0308 (4)	−1.0606 (13)	0.090 (3)	
H22	0.6893	0.0027	−1.0791	0.108*	
C23	0.5073 (14)	0.0361 (4)	−1.1541 (12)	0.094 (3)	
H23	0.4897	0.0119	−1.2379	0.112*	
C24	0.4124 (12)	0.0765 (4)	−1.1288 (12)	0.092 (3)	
H24	0.3301	0.0805	−1.1952	0.110*	
C25	0.4391 (10)	0.1116 (4)	−1.0039 (12)	0.080 (3)	
H25	0.3722	0.1388	−0.9847	0.095*	

### (RR-4) (1R*,2R*)-7-Methoxy-2-methyl-3-(4-phenylbutyl)-2,3,4,5-tetrahydro-1H-3-benzazepin-1-ol Atomic displacement parameters (Å^2^)

**Table:**

	*U*^11^	*U*^22^	*U*^33^	*U*^12^	*U*^13^	*U*^23^
C1	0.042 (3)	0.055 (4)	0.030 (3)	0.004 (3)	−0.002 (3)	0.008 (3)
C2	0.033 (3)	0.061 (4)	0.030 (3)	−0.007 (3)	0.001 (3)	0.007 (3)
N3	0.050 (3)	0.060 (4)	0.023 (2)	0.004 (3)	0.004 (2)	0.003 (2)
C4	0.061 (5)	0.063 (5)	0.030 (3)	0.007 (4)	−0.007 (3)	−0.001 (3)
C5	0.073 (5)	0.063 (5)	0.022 (3)	0.005 (4)	0.009 (3)	0.004 (3)
C6	0.047 (4)	0.051 (4)	0.027 (3)	−0.003 (3)	0.001 (3)	0.000 (3)
C7	0.057 (4)	0.060 (4)	0.025 (3)	0.001 (3)	0.000 (3)	0.001 (3)
C8	0.059 (4)	0.067 (5)	0.039 (4)	0.010 (4)	0.001 (3)	0.001 (4)
C9	0.073 (5)	0.080 (6)	0.033 (4)	0.014 (4)	0.001 (4)	0.000 (4)
C10	0.059 (4)	0.074 (5)	0.028 (4)	0.014 (4)	0.002 (3)	0.004 (3)
C11	0.043 (3)	0.061 (5)	0.024 (3)	−0.004 (3)	0.005 (3)	0.004 (3)
O12	0.050 (2)	0.072 (3)	0.032 (2)	0.007 (2)	−0.0035 (19)	0.013 (2)
C13	0.044 (4)	0.079 (5)	0.046 (4)	−0.005 (4)	0.004 (3)	0.001 (4)
O14	0.096 (4)	0.078 (4)	0.044 (3)	0.030 (3)	0.002 (3)	−0.007 (3)
C15	0.090 (6)	0.063 (5)	0.059 (5)	0.020 (5)	−0.014 (4)	−0.009 (4)
C16	0.049 (4)	0.069 (5)	0.043 (4)	0.004 (4)	−0.005 (3)	−0.012 (4)
C17	0.051 (4)	0.061 (5)	0.046 (4)	0.002 (3)	0.000 (3)	−0.002 (4)
C18	0.065 (4)	0.058 (5)	0.044 (4)	−0.011 (4)	0.003 (3)	0.003 (3)
C19	0.126 (9)	0.088 (7)	0.055 (5)	−0.024 (6)	0.027 (5)	−0.016 (5)
C20	0.082 (6)	0.063 (5)	0.040 (4)	−0.012 (4)	0.006 (4)	0.000 (4)
C21	0.073 (5)	0.096 (7)	0.062 (5)	0.001 (5)	0.006 (4)	0.004 (5)
C22	0.111 (8)	0.065 (6)	0.095 (7)	0.006 (6)	0.033 (6)	−0.016 (5)
C23	0.125 (9)	0.091 (7)	0.065 (6)	−0.030 (7)	0.012 (6)	−0.024 (6)
C24	0.097 (7)	0.114 (9)	0.065 (6)	−0.021 (7)	−0.017 (5)	0.002 (6)
C25	0.083 (6)	0.075 (6)	0.080 (6)	0.012 (5)	0.008 (5)	0.006 (5)

### (RR-4) (1R*,2R*)-7-Methoxy-2-methyl-3-(4-phenylbutyl)-2,3,4,5-tetrahydro-1H-3-benzazepin-1-ol Geometric parameters (Å, º)

**Table:**

C1—O12	1.429 (7)	C13—H13C	0.9700
C1—C11	1.509 (9)	O14—C15	1.410 (9)
C1—C2	1.545 (9)	C15—H15A	0.9700
C1—H1	0.9900	C15—H15B	0.9700
C2—N3	1.498 (7)	C15—H15C	0.9700
C2—C13	1.521 (9)	C16—C17	1.492 (9)
C2—H2	0.9900	C16—H16A	0.9800
N3—C4	1.477 (8)	C16—H16B	0.9800
N3—C16	1.486 (8)	C17—C18	1.535 (10)
C4—C5	1.525 (9)	C17—H17A	0.9800
C4—H4A	0.9800	C17—H17B	0.9800
C4—H4B	0.9800	C18—C19	1.524 (10)
C5—C6	1.531 (8)	C18—H18A	0.9800
C5—H5A	0.9800	C18—H18B	0.9800
C5—H5B	0.9800	C19—C20	1.507 (11)
C6—C11	1.387 (9)	C19—H19A	0.9800
C6—C7	1.395 (9)	C19—H19B	0.9800
C7—C8	1.408 (9)	C20—C21	1.374 (11)
C7—H7	0.9400	C20—C25	1.378 (11)
C8—O14	1.361 (8)	C21—C22	1.410 (13)
C8—C9	1.384 (9)	C21—H21	0.9400
C9—C10	1.369 (9)	C22—C23	1.338 (14)
C9—H9	0.9400	C22—H22	0.9400
C10—C11	1.397 (8)	C23—C24	1.365 (14)
C10—H10	0.9400	C23—H23	0.9400
O12—H12	0.8300	C24—C25	1.383 (13)
C13—H13A	0.9700	C24—H24	0.9400
C13—H13B	0.9700	C25—H25	0.9400
			
O12—C1—C11	110.8 (5)	H13B—C13—H13C	109.5
O12—C1—C2	106.2 (5)	C8—O14—C15	118.5 (6)
C11—C1—C2	113.8 (5)	O14—C15—H15A	109.5
O12—C1—H1	108.6	O14—C15—H15B	109.5
C11—C1—H1	108.6	H15A—C15—H15B	109.5
C2—C1—H1	108.6	O14—C15—H15C	109.5
N3—C2—C13	114.9 (5)	H15A—C15—H15C	109.5
N3—C2—C1	112.7 (5)	H15B—C15—H15C	109.5
C13—C2—C1	111.8 (5)	N3—C16—C17	116.4 (5)
N3—C2—H2	105.5	N3—C16—H16A	108.2
C13—C2—H2	105.5	C17—C16—H16A	108.2
C1—C2—H2	105.5	N3—C16—H16B	108.2
C4—N3—C16	109.7 (5)	C17—C16—H16B	108.2
C4—N3—C2	113.6 (5)	H16A—C16—H16B	107.3
C16—N3—C2	111.1 (5)	C16—C17—C18	112.7 (6)
N3—C4—C5	115.2 (5)	C16—C17—H17A	109.0
N3—C4—H4A	108.5	C18—C17—H17A	109.0
C5—C4—H4A	108.5	C16—C17—H17B	109.0
N3—C4—H4B	108.5	C18—C17—H17B	109.0
C5—C4—H4B	108.5	H17A—C17—H17B	107.8
H4A—C4—H4B	107.5	C19—C18—C17	111.9 (6)
C4—C5—C6	113.5 (5)	C19—C18—H18A	109.2
C4—C5—H5A	108.9	C17—C18—H18A	109.2
C6—C5—H5A	108.9	C19—C18—H18B	109.2
C4—C5—H5B	108.9	C17—C18—H18B	109.2
C6—C5—H5B	108.9	H18A—C18—H18B	107.9
H5A—C5—H5B	107.7	C20—C19—C18	113.8 (7)
C11—C6—C7	121.4 (5)	C20—C19—H19A	108.8
C11—C6—C5	120.2 (6)	C18—C19—H19A	108.8
C7—C6—C5	118.4 (5)	C20—C19—H19B	108.8
C6—C7—C8	119.8 (6)	C18—C19—H19B	108.8
C6—C7—H7	120.1	H19A—C19—H19B	107.7
C8—C7—H7	120.1	C21—C20—C25	117.9 (8)
O14—C8—C9	117.0 (6)	C21—C20—C19	119.6 (9)
O14—C8—C7	124.5 (6)	C25—C20—C19	122.6 (8)
C9—C8—C7	118.4 (7)	C20—C21—C22	119.6 (9)
C10—C9—C8	121.0 (6)	C20—C21—H21	120.2
C10—C9—H9	119.5	C22—C21—H21	120.2
C8—C9—H9	119.5	C23—C22—C21	121.0 (9)
C9—C10—C11	121.8 (6)	C23—C22—H22	119.5
C9—C10—H10	119.1	C21—C22—H22	119.5
C11—C10—H10	119.1	C22—C23—C24	120.4 (9)
C6—C11—C10	117.5 (6)	C22—C23—H23	119.8
C6—C11—C1	121.5 (5)	C24—C23—H23	119.8
C10—C11—C1	120.9 (6)	C23—C24—C25	119.1 (10)
C1—O12—H12	109.5	C23—C24—H24	120.5
C2—C13—H13A	109.5	C25—C24—H24	120.5
C2—C13—H13B	109.5	C20—C25—C24	122.0 (9)
H13A—C13—H13B	109.5	C20—C25—H25	119.0
C2—C13—H13C	109.5	C24—C25—H25	119.0
H13A—C13—H13C	109.5		
			
O12—C1—C2—N3	−155.6 (5)	C9—C10—C11—C6	−1.4 (10)
C11—C1—C2—N3	82.3 (6)	C9—C10—C11—C1	−178.8 (7)
O12—C1—C2—C13	73.2 (7)	O12—C1—C11—C6	177.6 (6)
C11—C1—C2—C13	−49.0 (7)	C2—C1—C11—C6	−62.8 (7)
C13—C2—N3—C4	62.0 (7)	O12—C1—C11—C10	−5.1 (8)
C1—C2—N3—C4	−67.7 (7)	C2—C1—C11—C10	114.5 (7)
C13—C2—N3—C16	−62.1 (7)	C9—C8—O14—C15	−177.4 (7)
C1—C2—N3—C16	168.2 (5)	C7—C8—O14—C15	0.6 (11)
C16—N3—C4—C5	−167.4 (5)	C4—N3—C16—C17	177.1 (6)
C2—N3—C4—C5	67.6 (7)	C2—N3—C16—C17	−56.6 (7)
N3—C4—C5—C6	−80.9 (7)	N3—C16—C17—C18	−166.4 (6)
C4—C5—C6—C11	63.5 (8)	C16—C17—C18—C19	76.1 (9)
C4—C5—C6—C7	−116.2 (7)	C17—C18—C19—C20	177.5 (7)
C11—C6—C7—C8	1.0 (10)	C18—C19—C20—C21	−98.4 (10)
C5—C6—C7—C8	−179.2 (6)	C18—C19—C20—C25	81.5 (11)
C6—C7—C8—O14	179.8 (7)	C25—C20—C21—C22	0.7 (12)
C6—C7—C8—C9	−2.2 (10)	C19—C20—C21—C22	−179.3 (8)
O14—C8—C9—C10	179.8 (7)	C20—C21—C22—C23	0.3 (14)
C7—C8—C9—C10	1.6 (11)	C21—C22—C23—C24	−0.3 (16)
C8—C9—C10—C11	0.2 (12)	C22—C23—C24—C25	−0.7 (16)
C7—C6—C11—C10	0.7 (9)	C21—C20—C25—C24	−1.7 (13)
C5—C6—C11—C10	−179.0 (6)	C19—C20—C25—C24	178.3 (8)
C7—C6—C11—C1	178.1 (6)	C23—C24—C25—C20	1.8 (15)
C5—C6—C11—C1	−1.7 (9)		

### (RR-4) (1R*,2R*)-7-Methoxy-2-methyl-3-(4-phenylbutyl)-2,3,4,5-tetrahydro-1H-3-benzazepin-1-ol Hydrogen-bond geometry (Å, º)

**Table:**

*D*—H···*A*	*D*—H	H···*A*	*D*···*A*	*D*—H···*A*
O12—H12···N3^i^	0.83	1.97	2.796 (6)	172
C15—H15*C*···O14^ii^	0.97	2.58	3.365 (9)	138

Symmetry codes: (i) −*x*+3/2, *y*, *z*+1/2; (ii) −*x*+1/2, *y*, *z*−1/2.
